# Periprosthetic joint infection caused by Brucella following total joint arthroplasty

**DOI:** 10.3389/fsurg.2025.1645618

**Published:** 2025-10-08

**Authors:** Wei Wang, Li Wang, Haiwei Dou, Olzhas Bekarissov, Habaxi Kaken

**Affiliations:** 1 Department of Joint Surgery, People’s Hospital of Xinjiang Uygur Autonomous Region, Urumqi, Xinjiang, China; 2 National Scientific Center of Traumatology and Orthopedics named after Academician N.D. Batpenov, Astana City, Kazakhstan

**Keywords:** Brucella, arthroplasty, infection, erythrocyte sedimentation rate (ESR), C-reactive protein (CRP)

## Abstract

**Background:**

Brucellosis is a common zoonotic infection that imposes a substantial economic burden on China, particularly in Xinjiang. This study aims to discuss the treatment of Brucella infection following total joint arthroplasty and evaluate its therapeutic effectiveness.

**Methods:**

We conducted a retrospective case series analyzing 8 patients who developed periprosthetic joint infection (PJI) due to Brucella after arthroplasty in our department between March 2009 and March 2019. The age range of these patients was 55–79 years, with an average age of 65.6 ± 1 year. Various parameters, including the Harris hip score (HHS), knee range of motion (ROM), visual analog scale (VAS) score for pain assessment, erythrocyte sedimentation rate (ESR), and C-reactive protein (CRP) level, were assessed before and after the revision surgery. Postoperative x-rays were used to assess the curative effect of revision surgery.

**Results:**

All patients experienced pain and elevated ESR levels. However, none of these patients exhibited deep vein thrombosis (DVT) or nerve damage. Additionally, no skin sinuses were detected. All infected patients underwent revision surgery subsequent to initial total joint arthroplasty. The follow-up period ranged from 6 to 30 months, with an average duration of 14 ± 0.5 months. After revision surgery, both HHS and Harris score assessments, as well as x-rays, were conducted to evaluate the curative effect. No cases of aseptic loosening or prosthesis fracture occurred during or after the revision operation, and no recurrence of infection were observed. The average knee ROM improved to 90 ± 3°.

**Conclusions:**

Recent clinical findings indicate that systemic antibiotic chemotherapy combined with surgical techniques is effective in treating patients following total joint arthroplasty. Revision surgery significantly improves joint function and alleviates pain.

## 1 Introduction

Brucellosis is a zoonotic disease caused by the gram-negative bacilli Brucella. From history, we know that Hippocrates may have described Brucella in his treatise “On epidemics,” and there is some evidence to prove that the disease was common in the ancient world (1). Brucellosis is a disease that primarily affects animals, such as cattle, goats, and pigs, but can also spread to humans. The disease is transmitted through direct contact with infected animals or the consumption of contaminated animal products, such as unpasteurized milk and cheese. Currently, it is still highly endemic in the western part of China. Although brucellosis is rare in developed countries, it remains a significant public health concern in many parts of the world, particularly in areas where there is a high prevalence of the disease in animals. In China, for example, brucellosis is considered one of the most important zoonotic diseases, with an estimated 100,000 human cases reported each year.

Total joint arthroplasty is a common surgical procedure used to treat various joint conditions, such as osteoarthritis and rheumatoid arthritis. However, the development of prosthetic joint infections, including those caused by Brucella, can lead to significant morbidity and mortality if not managed appropriately. Therefore, it is important to understand the clinical characteristics, diagnosis, and treatment options for patients with Brucella prosthetic joint infection. As arthroplasty is widely used, an increasing number of papers are reporting the use of arthroplasty in infected joints, such as tuberculosis joints (2, 3). Our hospital has 3200 beds for patients, including 150 beds for the orthopedics department and 15 beds for special infectious diseases, such as joint tuberculosis. On the basis of these clinical experiences, we carried out a revision operation for Brucella infection after total arthroplasty. The aim of this paper is to present our experience in managing patients with Brucella prosthetic joint infection following total joint arthroplasty. We hope that our findings contribute to the development of effective strategies for the prevention, diagnosis, and treatment of this challenging condition.

## 2 Materials and methods

This retrospective case series was approved by the People's Hospital of Xiniiang Uygur Autonomous Region Orthopedics Center. In accordance with China's brucellosis diagnosis and treatment guidelines (3), the diagnosis of brucellosis should be combined with an epidemiological history, clinical manifestations and laboratory examination. The diagnostic criteria for Brucella periprosthetic joint infection (PJI) included: (1) clinical signs of infection (e.g., joint pain, swelling, or fever); (2) elevated inflammatory markers (ESR > 30 mm/h and/or CRP  > 10 mg/L); (3) positive serological test for Brucella (standard tube agglutination test titre ≥1:160); and (4) positive Brucella culture from intraoperative tissue samples and/or positive PCR result from synovial fluid or tissue. Between March 2009 and March 2019, we retrospectively analyzed 8 patients who had one stage revision surgery for PJI caused by Brucella after arthroplasty. Patients with hip dysplasia, fractures secondary to tumors, Paget's disease, or metabolic bone disease were excluded from the study.

**Figure 1:**
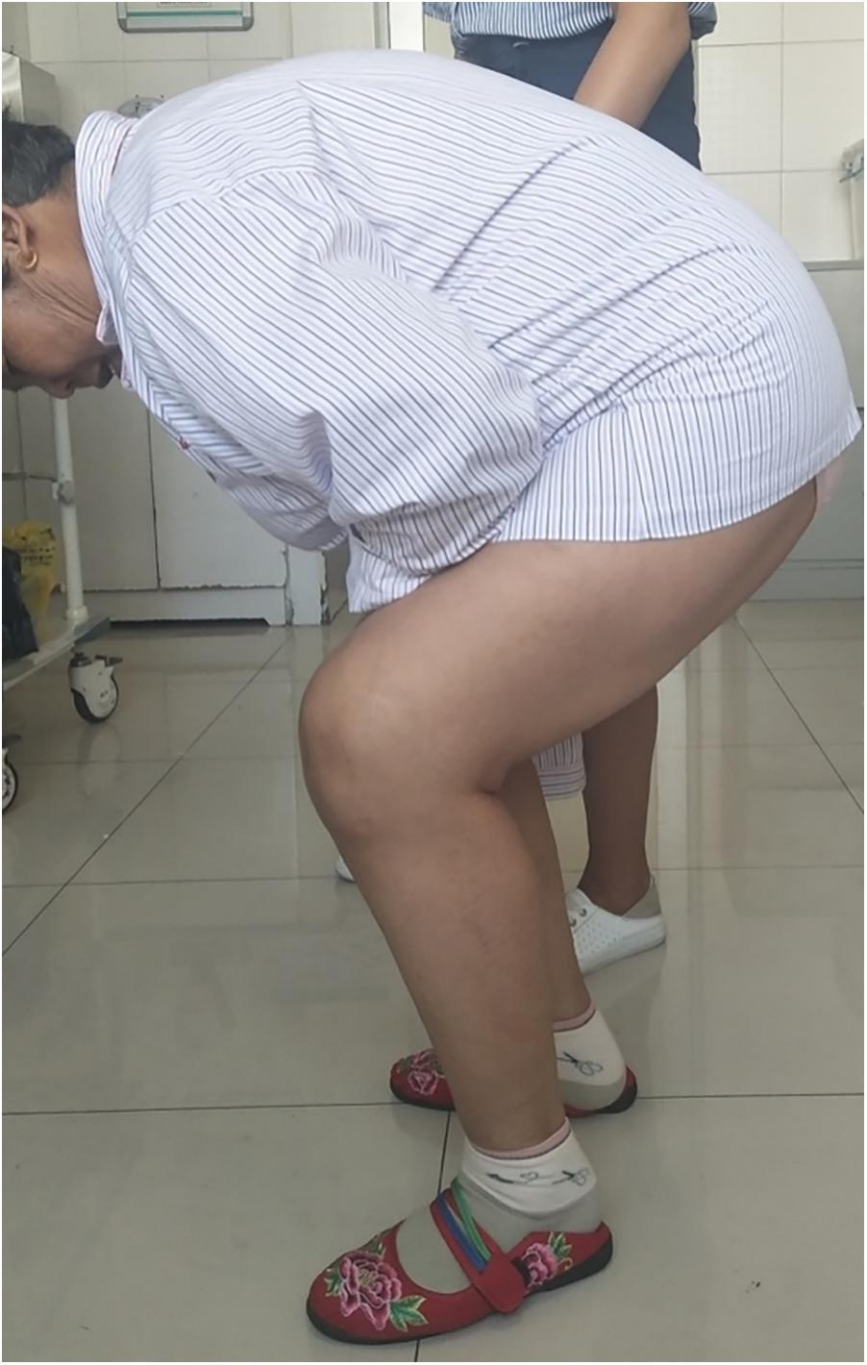
Patient have knee joint dysfunction pre-operation.

**Figure 2:**
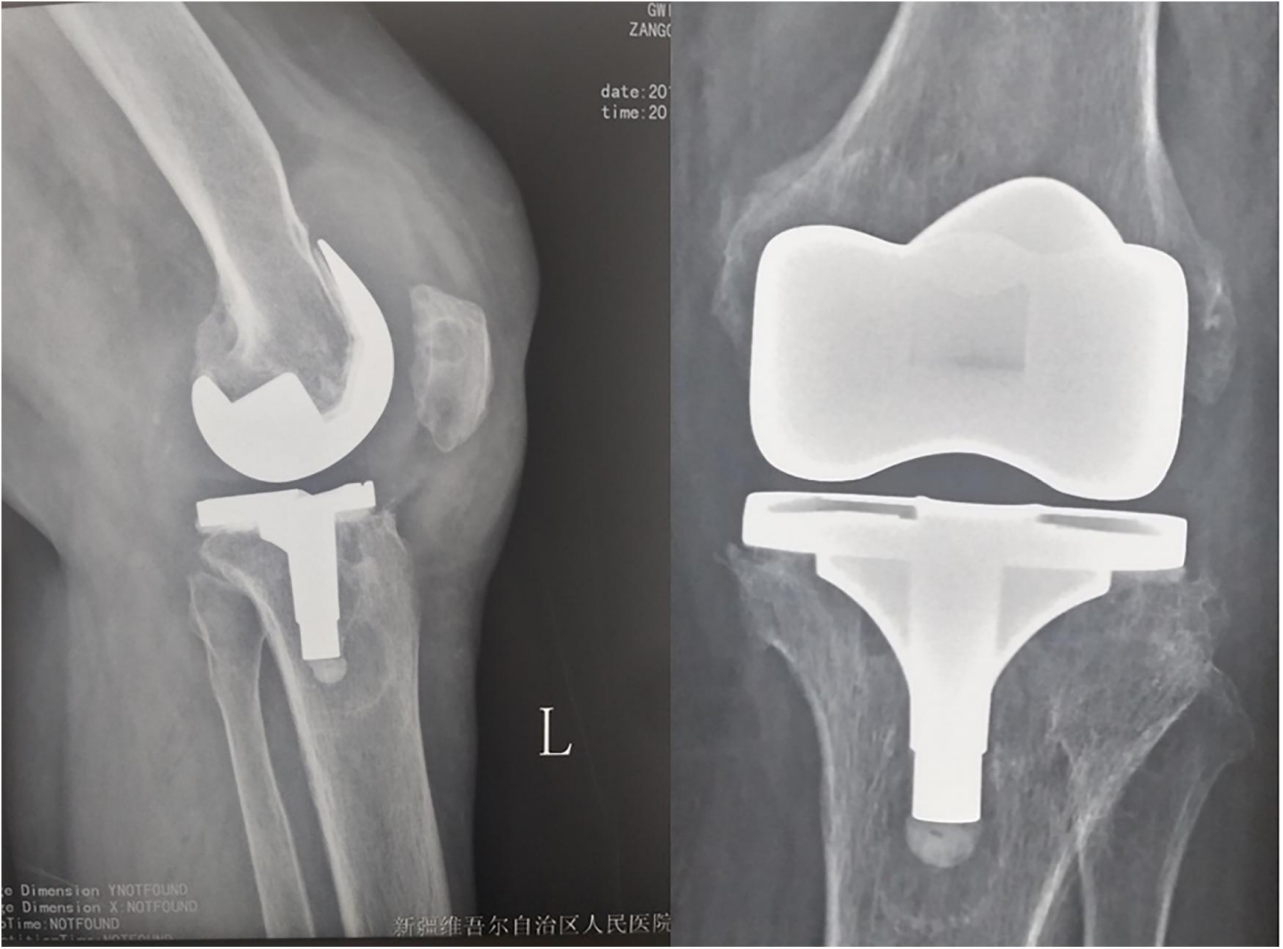
Pre-operation x-ray showed prosthesis loosing.

In our study, all of the cases were unilateral, five cases were male, and three cases were female. The patients' ages ranged from 55 to 79 years, with an average age of 65.6 ± 1 year. Six patients were herdsmen, and 2 lived in the city. On the basis of the clinical examination, we detected pain, joint swelling, joint dysfunction, and clinical weight loss, and 4 patients were febrile (Figure 1). One patient had swollen testicles and was previously treated in the urology department. On x-ray examination, we detected prosthesis loosening (Figure 2). From computed tomography (CT) scans, we found a low-density area indicating the formation of purulent joint tissue around the joints. Blood tests revealed an erythrocyte sedimentation rate (ESR) of 39–87 mm/h (normal 0–20 mm/h). All patients' C-reactive protein (CRP) levels were 12–112 mg/L (normal 1–8 mg/L) higher than normal. All patients had positive serological tests for Brucella (SAT titre ≥1:160). Intraoperative tissue cultures were positive for Brucella species in 7 cases (87.5%), and PCR analysis of synovial tissue confirmed Brucella infection in all 8 cases (100%). Anti-brucellosis treatment was started 2 weeks before surgery, and the medication regimen was doxycycline (100 mg, 2 times/d) combined with rifampicin (600 mg/d). All these findings suggest that preoperative nutritional support therapy is an important part of treatment. The VAS score was taken before and after the operation to assess the degree of pain. Two kinds of scores were used to evaluate the function. For knee revision patients, we used the HSS score. The preoperative HSS score ranged from 25–45 points (average 29.0 ± 2 points), the average ROM was 60 ± 5°, and the hip Harris score ranged from 30–50 points (average 36.5 ± 8.5 points). All patients were followed for 6–30 months; the average follow-up period was 14 ± 0.5 months.

**Figure 3:**
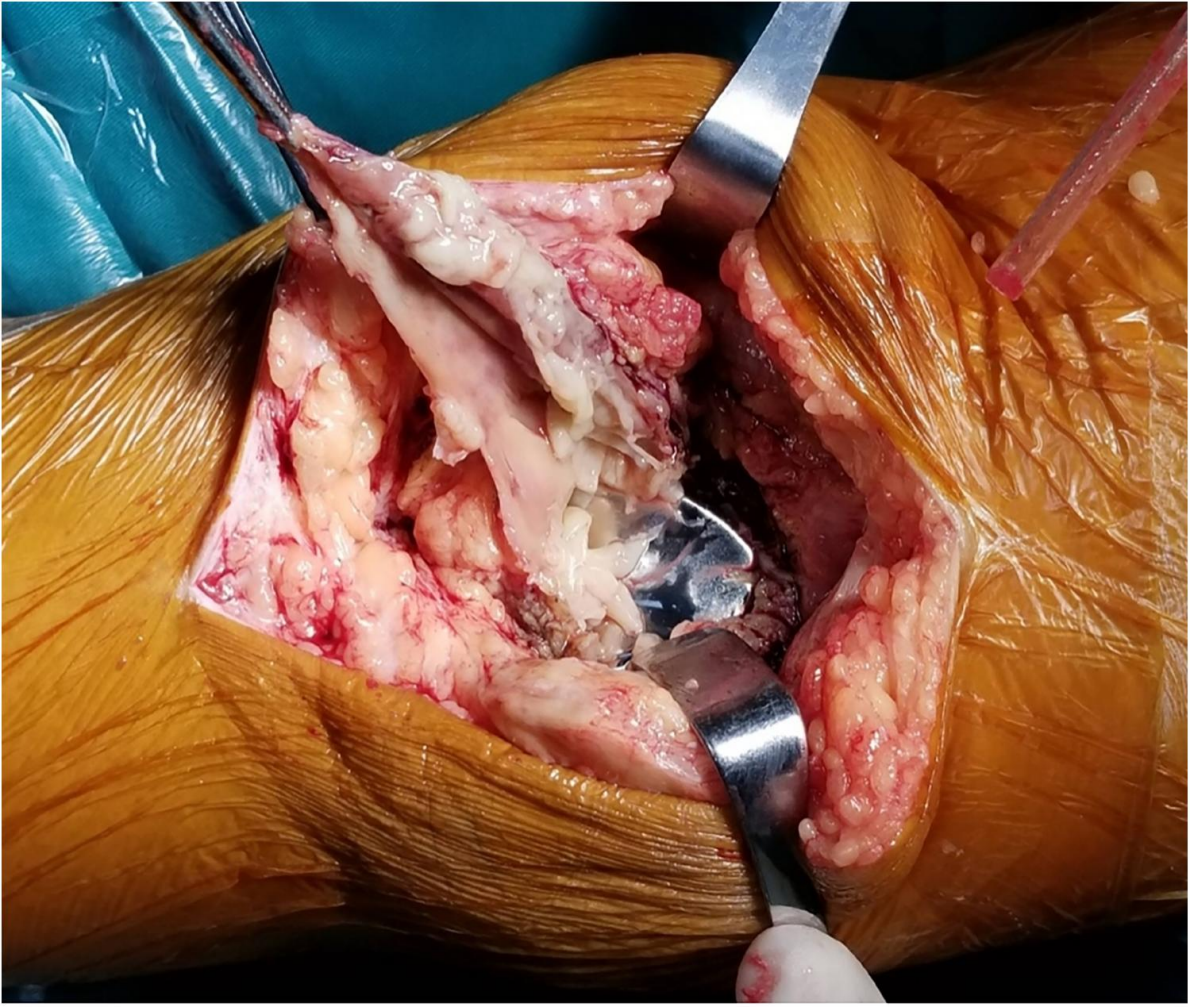
Synovial partial necrosis, purulent and necrotic tissue around prothesis.

**Figure 4:**
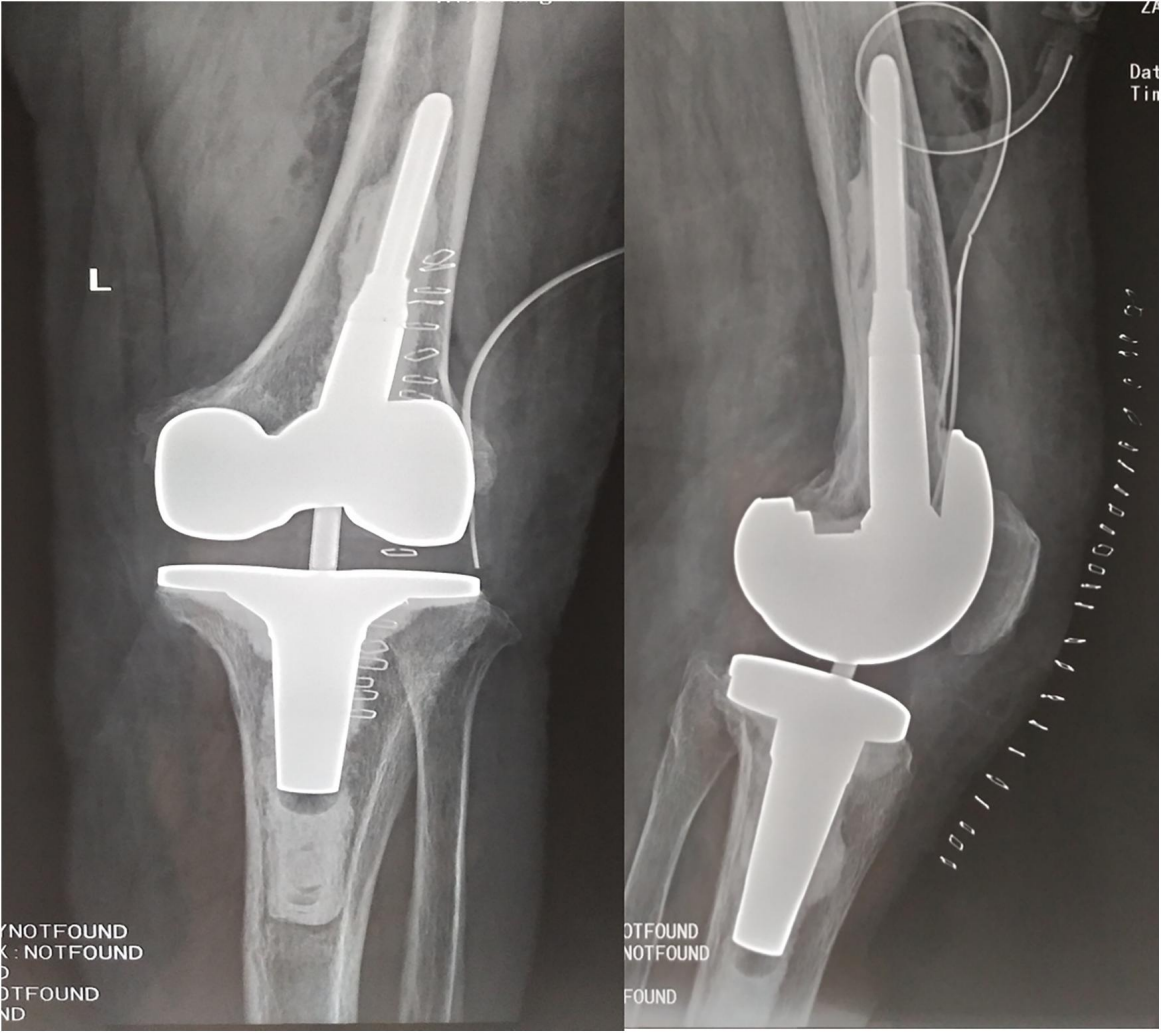
Post-operation x-ray shows no prothesis loosening happened, implants placed good position.

**Figure 5:**
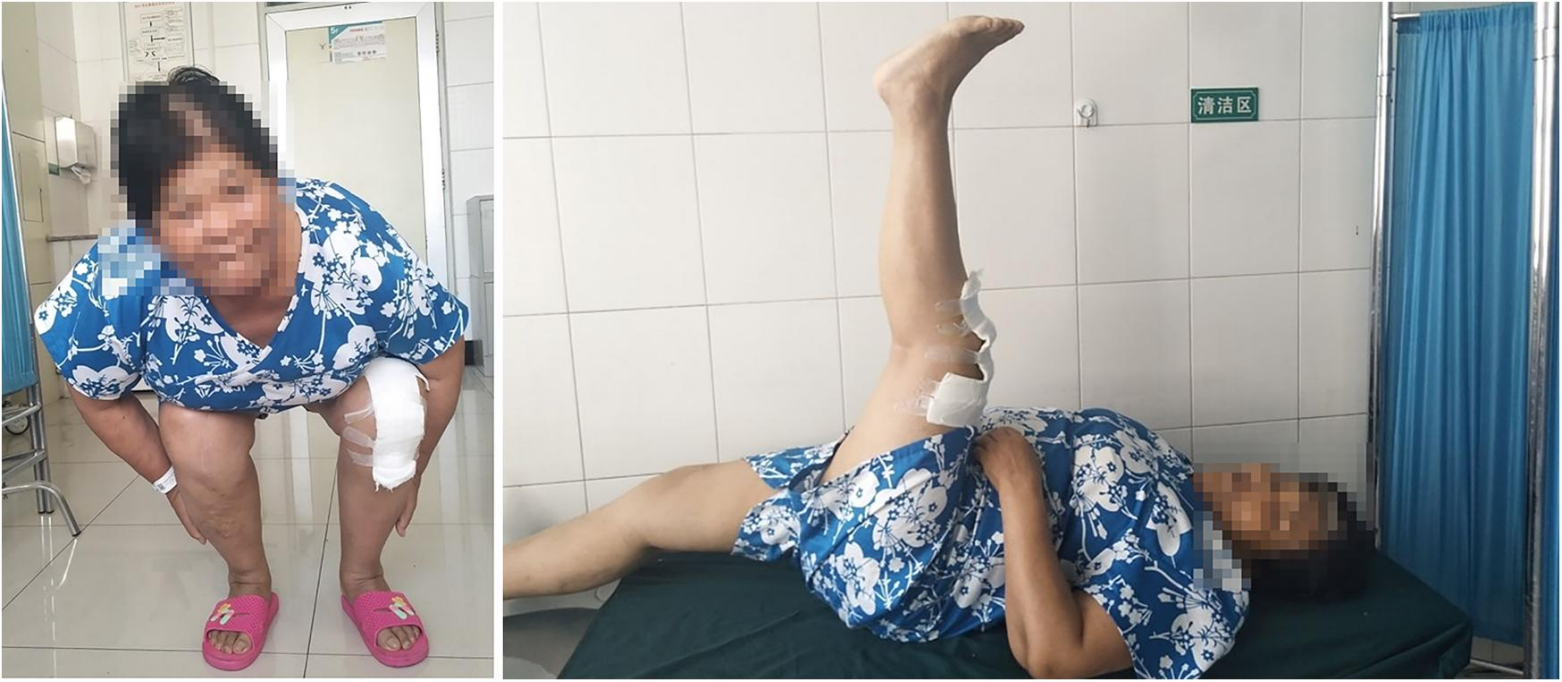
Patient has painless and good function knee joint post-operation.

Surgical procedures were performed under general anesthesia. For knee revisions, a standard medial parapatellar arthrotomy was used with a tourniquet. For hip revisions, a posterolateral approach was employed. During the operation, purulent or necrotic tissue around the prosthesis was observed. Purulent fluid was turbid and pale yellow, with synovial hyperplasia edema and synovial partial necrosis (Figure 3). We performed debridement first; pulse pulse lavage helped to eliminate Brucella bacillus. After this step, the gloves were changed, and a new operation instrument was used for revision. After debridement and installing the prosthesis, pulse lavage was performed after the bone cement was dry, and doxycycline powder (100 mg) was used directly around the prosthesis. No drainage tube was placed. The most conclusive means of establishing a diagnosis of brucellosis is positive cultures from normally sterile body fluids or tissues. During the operation, pus was cultured, and the tissues were removed and sent for routine pathological examination. Synovial fluid and tissue samples were collected for Brucella culture on specific media and PCR analysis using species-specific primers. Post-operation x-ray exam shows good position of prosthesis (Figure 4), and patients feel good also have good function (Figure 5).

Statistical analyses were performed using SPSS Statistics (version 19.0; IBM Corp., USA). The normality of continuous variables was assessed using the Shapiro–Wilk test. A two-sided p-value of <0.05 was considered statistically significant.

## 3 Results

### 3.1 Patient characteristics and surgical outcomes

As in Table 1, eight patients (5 males, 3 females; mean age 65.6 ± 7.2 years) underwent one-stage revision for Brucella PJI, with an equal distribution of hip and knee infections (*n* = 4 each). All patients presented with pain, and common clinical features included joint swelling and dysfunction. The mean time from initial arthroplasty to infection was 23.0 ± 12.5 months. All procedures were successfully completed without intraoperative complications. The mean operative time was 70 min (arrange from 50 to 90 min) with an average blood loss of 350 ml (arrange from 250 to 450 ml). All patients had successful outcomes, with no infection recurrence or implant failure observed.

**Table 1:** Demographic, clinical, and surgical characteristics of patients with Brucella periprosthetic joint infection.

Patient	Variable	1	2	3	4	5	6	7	8
Gender		Male	Female	Male	Male	Female	Female	Male	Male
Age		55	65	63	67	79	69	61	66
Joint pain		+	+	+	+	+	+	+	+
Weight loss		+	+	+	+	+	+	+	+
Febrile		+	−	+	−	−	−	+	+
Other organ		−	−	Testicle swollen	−	−	−	−	−
Bacterial culture		+	+	+	+	+	+	+	+
Pre-operation	CRP	46	39	57	78	87	86	82	78
	ESR	12	28	46	72	112	95	59	61
Post-operation	CRP	16	13	9	11	8	18	16	10
	ESR	10	16	15	21	26	28	19	23
Pre-operation	Harris score		30						50
	HSS score	25		25	26	26	45	27	
Post-operation	Harris score		86						91
	HSS score	84		86	82	83	89	82	
Pathological examination		+	+	+	+	+	+	+	+
DVT		−	−	−	−	−	−	−	-

Data are presented as raw values from the source table. The symbol “+” indicates presence, and “−” indicates absence of the condition.

The Harris and HSS scores were not available for all patients pre-operatively and post-operatively, as indicated in the original data.

CRP, C-reactive protein (mg/L); ESR, erythrocyte sedimentation rate (mm/h); HSS, hospital for special surgery score; DVT, deep vein thrombosis.

## 4 Discussion

Brucellosis is a zoonotic disease, and an increasing number of reports have shown that it is becoming more common in some undeveloped countries. Over 500 thousand cases of brucellosis are reported annually to the World Health Organization, and it is estimated that the annual number of losses from bovine brucellosis in Latin America is approximately US$ 600 million (4, 5). If the incidence of brucellosis is well controlled in animal reservoirs, there will be a corresponding and significant decline in incidence in humans. China implemented large-scale animal brucellosis prevention and control work in the 1960s and 1970s, which significantly reduced the prevalence of brucellosis; however, since the 1990s, the number of cases has risen significantly, especially in the western part of China. One study from our region (The Sixth People's Hospital of the Xinjiang Uygur Autonomous Region, hereafter referred to as the infection hospital) reported that a total of 2,041 patients with laboratory-confirmed brucellosis were admitted to the hospital between January 1st and December 31st, 2014, and the number of patients included was large (6).

Since the 1990s, an increasing number of arthroplasty procedures have been performed in China, leading to more reported cases of PJI have been reported. Although bacterial infections of prosthetic joints have been abundantly documented, Brucella infection following arthroplasty has rarely been reported; this infection was first described in 1991 in a Saudi woman with bilateral involvement caused by direct spread from knee abscesses (7).

One-stage or two-stage revision is still in the dispute phase in China. At present, bacterial biofilms are considered key factors in implant infection, and *in vitro* experiments have confirmed that the adhesion of brucellae to metals is lower than that of other bacteria. Studies have shown that we obtain good results in treating brucellae from spine infections and that internal fixation is safe because of good debridement. All these studies provide a reliable theoretical basis for prosthesis implantation in which we choose one-stage revision (8). We believe that one-stage revision combined with sufficient antimicrobial therapy throughout the disease course can relieve pain, improve joint function, and reduce the patient's hospitalization cycle and treatment costs. Thorough debridement uses a high concentration of rifampicin for washing before new implants are inserted. Up to 10% of patients relapse after antimicrobial therapy (9). This is a major challenge. The key point was debridement with pulse pressure washing, and the purulent and necrotic synovial tissue was clearly removed. In our study, we followed all patients for 6–30 months. x-ray revealed that no prosthesis loosening occurred. We allowed patients to walk as tolerated via a walker on the 1st day postoperation, and no DVT occurred. The VAS score was lower than the preoperative score, indicating that their pain was relieved. The average postoperative HSS score reached 80.7 ± 5.5, the average ROM improved to 90 ± 3°, and the average postoperative Harris score was 75.5 ± 0.5; thus, the preoperative score ranged from 30–50 points (average 36.5 ± 8.5 points).

The ESR and CRP level are also important in PJI infection diagnosis and treatment. Elevated CRP and increased ESR were the most common laboratory findings observed in our series, Buzgan et al. (10). reported similar results. The average ESR was 39–87 mm/H (normal 0–20 mm/H), and the average CRP level was 12–112 mg/L (normal 1–8 mg/L) preoperatively. Monitoring the effectiveness of anti-brucellosis treatment is crucial. If doxycycline (100 mg, 2 times/d) combined with rifampicin (600 mg/d) was used for 2 weeks but the ESR and CRP did not decrease, there was brucellal drug resistance, or if the patient had other kinds of infections, there was a high risk of recurrence. Therefore, we suggest the use of doxycycline and rifampicin for 2 weeks until there is a downward trend in the ESR and CRP levels, after which the operation can be performed. In our study, we used doxycycline (100 mg) powder directly around the prosthesis. Patients were encouraged to perform quadriceps strengthening exercises after they had recovered from anesthesia. All patients were allowed to walk on the 1st day postoperation. We found that from the 7th, 30th, and 90th days after the operation, the ESR and CRP level decreased and nearly reached normal values at 3 months, and the VAS score was significantly lower than that before the operation (*P* < 0.05). This means that surgery can help patients receive relief from pain and have good function, which is our aim. Previous reports have shown that arthroplasty has good results in treating joint brucellae infection.

There is convincing evidence that symptoms in brucellosis patients are protean and nonspecific (6, 11). Brucella organisms may localize to almost any organ, bone, lung, testis or liver. In this study, 1 patient had swollen testicles and was previously treated in the urology department. A study performed in Iran (12) revealed that pain, joint swelling, joint dysfunction, and clinical weight loss were common, and nearly 87% of patients experienced these conditions, which is similar to the findings of our study. Hepatosplenomegaly and lymphadenopathy may be found as well as signs and symptoms associated with other infected organs, but this was not found in our study.

Few cases have been reported in the published literature, and the treatment of Brucella PJI is equally challenging. Previous meta-analyses have reached different conclusions regarding the preferred regimens for treating brucellosis. Generally, dual or triple regimens are advisable. In 1986, the World Health Organization recommended the use of doxycycline in combination with rifampin for 6 weeks as the preferred treatment for adult acute brucellosis. In accordance with China's Practice Guidelines for brucellosis diagnosis and treatment (3), the use of doxycycline (100 mg every 12 h) and rifampin (600–900 mg every 24 h) was suggested. The recovery rate of human brucellosis has been reported to be between 80.6% in Farazi AA studies (13), but the prognosis of Brucella joint infection in many patients has not been reported. The use of an aminoglycoside with doxycycline is believed to reduce relapse, but we did not use this agent. Another reason for choosing rifampin is that many patients in the western part of China also have the same risk for tuberculosis (6, 8), and misdiagnosis of tuberculosis as brucellosis might lead to the inadvertent use of rifampin, which is almost guaranteed to result in rifampin resistance. Patients on doxycycline for brucellosis may have developed the Jarisch–Herxheimer reaction reported by John S (14), which did not occur in this study. Additionally, in our case series, the clinical results indicated that the treatment was satisfactory and safe and did not present obvious operation-related complications. Relapses most commonly occur within the first 6 months after the completion of therapy. We followed-up for 6–30 months with no evidence of disease recurrence or relapse. Antimicrobial therapy was continued for 12–24 weeks when symptoms were completely resolved and inflammatory biomarkers were normalized.

Similar to previous studies (5, 15), Brucella PJI in our cohort predominantly presented with localized symptoms and occurred long after initial arthroplasty. All our patients successfully underwent one-stage revision with doxycycline and rifampin combination therapy, showing no recurrence at mean 14-month follow-up. This contrasts with some earlier recommendations for two-stage revision (5) but aligns with emerging evidence supporting combined surgical and medical management (15). The diagnostic challenges we observed, particularly the need for multimodal confirmation through serology, PCR, and culture, further resonate with reports from both endemic and non-endemic regions (15), highlighting the universal complexities in managing this rare infection.

This study has several limitations that should be considered when interpreting the results. First, the small sample size of only 8 cases over a 10-year period inherently limits the statistical power and generalizability of our findings. Second, the retrospective, single-center design introduces potential selection and information biases, and the absence of a control group prevents definitive causal inferences regarding the efficacy of the treatment strategy. Additionally, the variation in follow-up duration may not fully capture long-term outcomes such as late recurrence or implant failure. Despite these limitations, this case series provides valuable preliminary insights into the management of a rare but serious complication, and future prospective, multi-center studies with larger cohorts and longer follow-up are warranted to validate our conclusions.

## 5 Conclusions

Revision for the treatment of Brucella infection following total joint arthroplasty is rare in China and abroad, and further in-depth research and long-term follow-up reports are needed. Systemic antibiotic chemotherapy and surgical techniques are both important treatments. Revision operations can improve patient efficacy, safety and patient satisfaction.

## Data Availability

The original contributions presented in the study are included in the article/Supplementary Material, further inquiries can be directed to the corresponding author.
